# Pharmaceutical Application of Tablet Film Coating

**DOI:** 10.3390/pharmaceutics12090853

**Published:** 2020-09-08

**Authors:** Ki-Soo Seo, Rajiv Bajracharya, Sang Hoon Lee, Hyo-Kyung Han

**Affiliations:** 1 College of Pharmacy, Dongguk University-Seoul, Dongguk-ro-32, Ilsan-Donggu, Goyang 10326, Korea; kisoo.seo@dong-wha.co.kr (K.-S.S.); rajivbajra@hotmail.com (R.B.); sh_lee@dongguk.edu (S.H.L.); 2 Research Institute, Dong Wha Pharm., Tapsil-ro-35, Giheung-gu, Yongin 17084, Korea

**Keywords:** film coating, active coating, modeling, coating uniformity, tablet

## Abstract

Tablet film coating is a common but critical process providing various functionalities to tablets, thereby meeting diverse clinical needs and increasing the value of oral solid dosage forms. Tablet film coating is a technology-driven process and the evolution of coated dosage forms relies on advancements in coating technology, equipment, analytical techniques, and coating materials. Although multiple coating techniques are developed for solvent-based or solvent-free coating processes, each method has advantages and disadvantages that may require continuous technical refinement. In the film coating process, intra- and inter-batch coating uniformity of tablets is critical to ensure the quality of the final product, especially for active film coating containing active pharmaceutical ingredients in the coating layer. In addition to experimental evaluation, computational modeling is also actively pursued to predict the influence of operation parameters on the quality of the final product and optimize process variables of tablet film coating. The concerted efforts of experiments and computational modeling can save time and cost in optimizing the tablet coating process. This review provides a brief overview of tablet film coating technology and modeling approaches with a focus on recent advancements in pharmaceutical applications.

## 1. Introduction

Tablets are among the most convenient and preferred oral dosage forms because of their many advantages, including ease of administration, high patient compliance, and cost-effectiveness. Among the multiple steps in pharmaceutical manufacturing of tablets, coating is a critical process that is often used for functional and aesthetic reasons [1]. Among three types of tablet-coating processes (sugar coating, film coating, and press coating), film coating is the most widely used approach to solve various issues encountered during manufacturing, transport, storage, and clinical use of drug products. For example, tablets containing active pharmaceutical ingredients (APIs) sensitive to light, oxidation, or moisture can be protected by film coating, leading to increased stability of drug products during manufacturing and storage. In addition, film coating can control the drug release patterns of tablets in terms of site, rate, and time [2,3,4,5]. Film coating is also applicable to mask taste and improve patient compliance. Accordingly, tablet film coating is widely used to achieve various pharmaceutical and therapeutic goals.

Conventional solvent-based film coating involves deposition of a thin polymer film on the surface of the tablet core, typically using a spray method. The coating solution or suspension contains polymers and other ingredients such as pigments and plasticizers, which is sprayed onto a rotating tablet bed inside a pan [6]. The drying process is accomplished by passing hot air through the tablet bed and permits removal of the solvent to leave a thin film on the surface of each tablet core [6]. Film formation depends on the physicochemical properties of polymers [7]. Plasticizers are also used to reduce the glass transition temperature (Tg) and increase flexibility to avoid cracking and subsequent peel-off of polymer films [7]. Many coating techniques have been developed for solvent-based or solvent free processes to improve the efficiency of the coating process. However, each method has its own advantages and disadvantages and may require continuous technical refinement. Tablet film coating is a technology-driven process, and the evolution of coated dosage forms depends on advancements in coating technology, equipment, analytical techniques, and coating materials.

In film coating processes, the intra- and inter-batch coating uniformity of tablets is critical to ensure the quality of the final product. This is especially important for active film coating with an active pharmaceutical ingredient in the coating layer. Modeling approaches are also actively pursued to predict the influence of operation parameters on the quality of final products and optimize the process variables of tablet film coating [8]. The concerted efforts of experiments and computational modeling can save time and cost to optimize the tablet coating process.

This review covers a brief overview of tablet film coating technology and modeling approaches, with a focus on recent advancements in pharmaceutical applications.

## 2. Film Coating Methods

Organic solvent-based film coating: Although the use of organic solvent is not preferred, film coating with hydrophobic or lipophilic polymers requires the use of organic solvents due to the low aqueous solubility of coating materials. The highly hydrophobic polymers are beneficial as moisture-protective coating polymers as they can reduce the water vapor permeability of the final film by preventing the movement of water molecules [9]. In addition, coating process using organic solvents can also reduce drug degradation by hydrolysis. Therefore, organic solvent-based coating is useful for moisture sensitive drugs. The rate of evaporation of the solvent is crucial for the quality of final product and many variables including temperature, atmospheric pressure, and air movement should be adjusted to optimize the evaporation rate [9]. Despite of pharmaceutical needs, organic solvent-based coating has many critical limitations because of potential toxicity of residual solvents, flammability, and environmental safety issues [10]. Even with a proper ventilation facility, it is difficult to completely remove organic solvent vapors from the coating room, increasing the risk of toxicity and explosion. Environmental and regulatory issues can increase the production costs.

Aqueous film coating: Aqueous coating is a widely used film coating method in current pharmaceutical practice. It has many advantages over organic solvent-based coatings in terms of operator safety, environmental pollution, and risk of explosion. Despite these benefits over organic solvent-based coating methods, aqueous coating also possesses certain drawbacks including energy- and time-consuming water evaporation process, longer processing time, validation of coating dispersion to control microbial presence, and potential activity loss of certain drugs caused by water or high coating temperature [11,12]. In addition, preparation of an aqueous coating solution with water-insoluble polymers requires the addition of a suitable suspending agent or plasticizer for a homogeneous coating solution [10]. Although aqueous film coating has some limitations, it can avoid the safety issues associated with organic solvent-based coating, and thus it is still widely used in the pharmaceutical industry. There are continuous efforts to reduce the processing time and improve productivity by process automation, process validation, and development of more efficient equipment, such as side-vented perforated coating pans and fluidized bed equipment [13].

Solvent-free coating: The use of solvents and heat exposure in the coating process can increase product instability, processing cost, and risk of environmental and safety hazards [14]. Therefore, solvent-free coating methods have been actively pursued to overcome the drawbacks of solvent-based coating. Solvent-free coating can reduce the process time and cost by avoiding expensive and time-consuming processes of solvent disposal [12]. Furthermore, since it does not require drying in most cases, solvent-free coating is applicable to heat-sensitive drugs [12]. Solvent-free coating includes various technologies such as compression coating, dry powder coating, electrostatic spray powder coating, and photocuring coating [10,15,16]. Recently, injection molding coating and hot melt coating have also been proposed as dry coating processes that do not require solvent [14,17,18,19]. In the injection molding coating process, the upper and lower surfaces of tablets are coated in two steps using a vertical injection molding unit. It is important to choose the right polymer because quality varies depending on workplace humidity and polymer properties. As high temperature (over 80 °C) is used for coating, it is important to examine the surface conditions of coated tablets after cooling [14,17]. Hot-melt coating is also a dry coating method in which a lipid excipient is heated for melting and then sprayed onto the surface of tablets to form a coherent coating layer [18]. This method has advantages including a short process time and no chemical interactions between the tablet ingredients and the inert coating materials. However, the high process temperatures depending on the melting point of coating materials may affect the stability of the tablet ingredients [20]. Like injection mold coating, many variables may occur depending on excipient characteristics [18,19]. Spray congealing or spray cooling is also a melting-based method that transforms a melt into spherical solid particles. This method has advantages including the absence of solvent, low cost, applicability to hygroscopic and water-sensitive substances, and the ability to obtain spherical free-flowing microparticles for tableting or capsule filling without the need of other downstream processes [21,22]. Spray congealing technology includes three steps (feed, atomization, and solidification stages) [23]. The first step involves the preparation of the fluid consisting of the molten carrier and drugs. As drugs may be dissolved or dispersed into the molten carrier, it is important to keep the fluid homogeneous for a uniform drug loading. During the atomization step, the molten fluid stream breaks up into small droplets that are quickly solidified upon cooling to produce the solid microparticles. In this process, viscosity is a critical factor deciding the viability of spray congealing and the size of produced microparticles [21,22]. In general, spray congealing is not suitable for highly viscous molten mixtures that may clog the feed tube or atomizer [22].

Although solvent-free coating may overcome some issues associated with solvent-based coating, the requirements for specific coating conditions, equipment, and coating materials limit wide application of solvent-free coating in the pharmaceutical industry [11].

## 3. Process Parameters and Factors Affecting Film Coating Quality

In the conventional solvent-based pan coating process, tablets are filled into the coater that is generally operated in batch mode and the batch size is determined by the mass of tablets from the compression unit operation [24]. During the process operation, the rotating pan allows tablets to circulate along the coater walls. When tablets pass the spray zone, the droplets of coating solution are deposited on the surface of tablets to form a coating film. At each cycle, tablets pass the spray zone with different orientation and thus the surfaces of tablets facing to the spray gun are frequently changed, leading to the entire surface coating of tablets [24]. After drying by a combination of hot airflow supplied from the upper region of the coater and conduction from the heated tablet bed, the obtained film must be smooth and uniform. However, the complexity of the coating process often leads to the coating defects including bridging, cracking, and orange-peel roughness [25,26]. These defects are mainly because process parameters are not satisfactory. Therefore, optimization of coating formulations (compositions), process variables, and equipment parameters are critical to obtain the uniform and smooth coating layer [27,28]. Some of process parameters and factors affecting film coating quality are discussed below.

**Figure 1:**
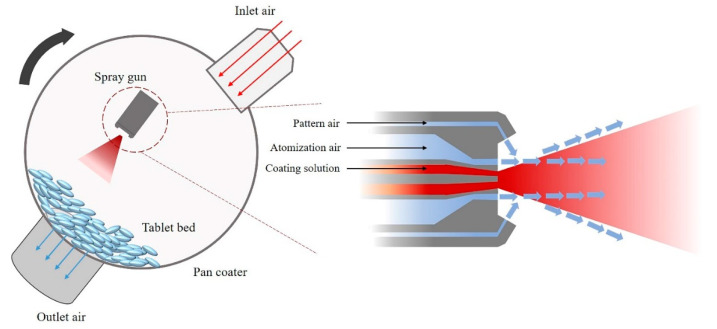
Simplified illustration of a coating pan.

Spray air flow rate: An important step in the coating process is spraying the coating solution. High-pressure atomization air disintegrates the coating solution into droplets that are pushed through the spray nozzle. If spraying only with atomization air, coating solution droplets may move directly into a narrow area of the tablet surface, which may cause problems with coating uniformity [29,30]. Therefore, both atomization and pattern air must act simultaneously to spray droplets homogeneously and widely (Figure 1). The ratio between atomization air and pattern air is an important parameter for coating efficiency. Atomization air and pattern air ratios closer to 1:1 correlate to smaller mean droplet size and better coating efficiency [31,32].

Spray rate: Along with the aforementioned spray air, another important parameter that affects the coating process is spray rate. As spray rate increases, droplet size increases and droplet velocity decreases. Spray rate affects coating quality not only as an individual parameter but also as a composite parameter with atomization air and pattern air [30]. Because mean droplet size does not change significantly when the ratio of atomization air and spray rate is fixed, the atomization air/spray rate ratio is one of the most important parameters that affect droplet size and coating quality [31,32]. In addition, droplet size determined by spray rate and atomization air is an important factor that determines the drying capacity in the coating process [33].

Inlet air/Outlet air: Sprayed droplets of coating solution are transferred to the tablet surface and dried to form a coating film. Heat energy must be supplied to dry the coating pan via heated inlet air flow. The relative humidity of supplied inlet air can have a significant impact on the related humidity of the coating pan and outlet air, which in turn affects drying efficiency [34]. In cases of over-drying due to excessive inlet air supply, the coating droplet may be dried before it adheres to the tablet surface. This may generate a large number of polymer particles, resulting in a rough surface of the coated tablet. Conversely, if sufficient inlet air is not supplied for drying, twinning and tablet agglomeration may occur due to the viscosity of droplets on the wet tablet surface [30]. Outlet air (exhausted air) temperature can indirectly indicate the temperature of the coating pan or tablet during the coating process. Generally, tablet temperature inside the coating pan is ~2–3 °C lower than the outlet air temperature [30,35]. Outlet air temperature should be changed according to the characteristics of the solvent or spray rate. Inlet and outlet air temperature can be controlled according to the coating machine brand.

Droplet size: Droplet size is highly affected by characteristics of the coating solution and process conditions. It also has a high correlation with coating efficiency. As the ratio of atomization air to pattern air approaches 1:1, droplet size becomes smaller and coating efficiency improves [31,32]. In general, when droplet size decreases, a homogenous film layer is formed on the surface of tablets. Conversely, if the size of the droplet in contact with the tablet surface is too large, tablet surface roughness may increase. Droplet size is highly dependent on process conditions (spray air flow rate, spray rate, gun-to-bed distance, viscosity, etc.) and requires careful control.

Solid content and viscosity: The amount of polymers in the coating solution is important in determining the viscosity of coating solution. The viscosity of coating solution is increased by using a high molecular weight polymer or high polymer content in coating solution [36]. High solid content in coating solution increases tablet weight faster but can lead to difficulty transferring a viscous coating liquid. If necessary, the coating solution may be heated to raise the temperature and lower the viscosity [30,37]. Conversely, coating solution with low solid content contains more moisture, increasing the relative humidity inside the coating pan and resulting in poor drying efficiency. In this case, inlet air flow rate or inlet air temperature must be optimized.

Gun-to-bed distance: Gun-to-bed distance represents the distance between the virtual plane of the tablet mass inside the coating pan and the tip of the spray gun nozzle, which can often be changed by an operator’s subjectivity [34]. As the droplets of coating solution move away from the nozzle tip, droplet velocity decreases and agglomeration occurs, resulting in increased droplet size (diameter). As the gun-to-bed distance increases, coating process efficiency decreases. Droplets may be dried before they reach the tablet surface, resulting in a rough tablet surface [37]. Conversely, if the gun-to-bed distance is short, sprayed droplets adhere to the tablet surface before they are dried and the tablet surface gets wet. Wet tablet surfaces can cause twinning or coating surface dissolution [34]. Tablet surface moisture should be controlled by considering the aforementioned factors including coating machine size.

Curing time: Coating process is not necessarily finished when the coating solution sprayed onto the tablet reaches the target tablet weight. After spraying, the coating layer contains a small amount of undried solvent. After the solvent evaporates, the polymer coalesces to form a dense structure. Post-coating thermal treatment (curing) induces residual solvent drying and polymer coalescence [38,39]. Curing time usually lasts from 1 to several hours. The dissolution profile may change with curing because the coating layer hardens [40,41].

More details on various factors affecting the coating quality and the underlying mechanisms have been reviewed elsewhere [28,29,30].

## 4. Pharmaceutical Application of Film Coating

### 4.1. Modified Drug Release

In many cases, modified drug release is beneficial for improving drug efficacy and patient compliance or prolonging the duration of action [42]. Therefore, tablet film coating with various polymers is actively pursued to achieve modified drug release by controlling the rate and/or sites of drug release. Representative film coating approaches for modified drug release are discussed below.

#### 4.1.1. Delayed Drug Release

The main advantage of enteric coating is increasing drug stability in the harsh gastric environment and/or reducing undesirable gastric irritation caused by drugs. To prevent premature drug release in the stomach and ensure drug release mainly in the small intestine, polymers with pH-dependent solubility or water-insoluble polymers are applied to enteric film coating. These polymers can be used alone, in combination, or one after the other to ensure delayed drug release. Proton pump inhibitors including rabeprazole, pantoprazole, omeprazole, esomeprazole, and lansoprazole are acid-labile and require an enteric coating formulation to increase drug stability in the stomach. Enteric coated tablets of esomeprazole have been developed using various polymers such as Eudragit^®^ L-30 D-55, hydroxypropyl methylcellulose phthalate, cellulose acetate phthalate, and Acryl-EZE^®^ [43,44]. Similarly, Gobinath et al. [45] developed enteric coated tablets of pantoprazole (which also irritates the gastric mucosa to cause nausea and vomiting) with cellulose acetate phthalate (CAP) and Eudragit^®^ L100. Tirpude and Puranik [46] demonstrated better performance of rabeprazole with dual delayed-release enteric coating using two different enteric polymers: an inner acrylic coating followed by an outer cellulose coating [46]. Time-dependent drug release can also be achieved via preparation of two different enteric-coated granules, one that dissolves in the upper intestine and the other that dissolves in the lower intestine. The Food and drug administration (FDA) has approved a dual delayed-release formulation of dexlansoprazole comprised of two types of enteric-coated granules with different pH-dependent dissolution profiles, one released at 1–2 h after dosing and the other released at 5–6 h after dosing [47,48]. This formulation in a once-daily dosing is effective to extend drug absorption and control intragastric acidity for longer periods of time [47,48].

Physicochemical instability and low permeability in the gastrointestinal (GI) tract limit oral delivery of macromolecules, such as proteins and peptides. Among various strategies to overcome these absorption barriers and low bioavailability of macromolecules, enteric coating is the most actively pursued strategy alone or in combination with other approaches [49]. Wong et al. [50] developed an enteric coated-insulin tablet composed of chitosan (absorption enhancer), sodium glycocholate (enzyme inhibitor), and an enteric coating layer with cellulose acetate hydrogen phthalate. This enteric coated tablet displayed minimal insulin release at acidic pH and effectively increased insulin-dependent Glut-4 translocation [50]. Similarly, many other insulin formulations for oral delivery use enteric film coating. There are also enteric coated formulations of enzymes (e.g., pancreatin and lactase) or hormones in the market or under clinical investigation [49,51].

Film coating for delayed drug release has also been attempted for colon-targeted drug delivery or chronotherapeutic drug delivery matched with circadian rhythms. Applications of enteric film coating for pulsatile drug release are discussed below.

**Figure 2:**
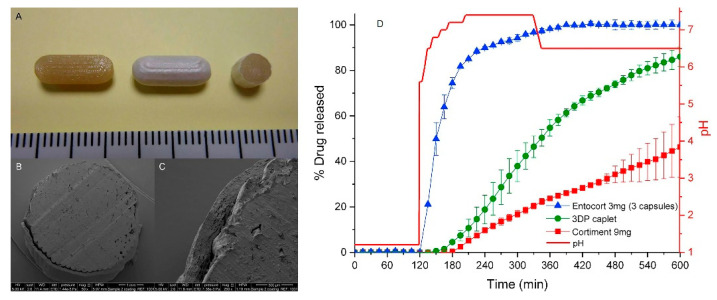
Images of 3DP fabricated caplets (**A**) from left to right: caplet prior to coating, caplet after coating and cross section of coated caplet (scale in cm). (**B**,**C**) SEM images of internal structure of cross section of a coated caplet. (**D**) Drug release from two commercial products (Cortiment^®^, Entocort^®^) and the coated caplets (3DP caplet) in 0.1 M HCl for 2 h followed by physiological bicarbonate buffer under dynamic pH conditions (pH 5.6–7.4 and then 6.5) controlled by the Auto pH System TM. Red line shows the real-time pH values. Figures were adopted with permission from [61], Elsevier, 2015.

Colon-targeted drug release: Colon-targeted drug delivery systems are required for local treatment of colon-specific diseases such as Crohn’s disease, irritable bowel syndrome (IBS), and colon cancer [2,52,53]. In addition, there is great interest in colonic drug release as an effective approach for improving the bioavailability of peptides and protein drugs [2]. As (i) the GI tract undergoes dynamic changes in motility, enzymatic activity, fluid content, and pH from the stomach to the intestine, and (ii) the pathologic condition of a disease site in the colon is markedly different from normal and healthy regions [2], surface coating for colon targeted drug release is more complex than enteric coating targeting the small intestine. Various film coating approaches have been explored for colonic drug delivery, including pH-dependent film coating, enzymatically degradable coating, and time-dependent film coating [53,54]. However, most of these systems exhibit suboptimal drug release profiles. Therefore, dual coating technology based on different release-triggering mechanisms is actively pursued to improve colon specific delivery. Ibekwe et al. [55] reported a novel colonic coating technology that integrated pH-dependent and bacterially triggered systems into a single layer matrix film. Tablets were coated using a pH-dependent polymer and natural polysaccharide (enzyme-sensitive component) that work in a complementary manner to facilitate site-specific release [56]. Recently, Dodoo et al. [57] also demonstrated the effectiveness of this dual-trigger coating technology in colonic delivery of probiotics. To improve the effectiveness of pH-dependent systems, ColoPulse technology has also been developed by incorporating super-disintegrants into the coating matrix [2]. Previous studies have demonstrated the effectiveness of ColoPulse technology for drug delivery to the ileo-colonic region of patients with Crohn’s disease [58,59,60]. Goyanes et al. [61] developed a controlled-release tablet for the colonic delivery of budesonide. Capsule-shaped tablet (caplet) containing 9 mg budesonide was fabricated by using 3D printing technology and then was coated with Eudragit^®^ L100 in spray fluidized bed coater. The surface and cross section images of caplets were obtained by using a scanning electron microscope (SEM), displaying the outer coating layer (Figure 2). Drug release profiles of the coated caplets were also examined in a dynamic dissolution buffer system simulating gastrointestinal conditions. As shown in Figure 2D, the coated caplet was resistant to acidic conditions and exhibited a pH-dependent drug release profile. It started drug release after 1 h in the small intestinal condition and then continued drug release in a sustained manner throughout the conditions of distal intestine and colon [61].

Chronotherapeutic drug release: Release of APIs can be delayed for a programmable period of time to meet the chronotherapeutic needs, in particular for circadian symptoms [62,63]. Chronic diseases with circadian symptoms that likely recur in the night or early morning include cardiovascular disease, bronchial asthma, rheumatoid arthritis, and sleep disorders. Although drugs are administered at bedtime, pulsatile drug release matched with the circadian rhythms of the disease can selectively cover the critical period of the disease without requiring the patient to wake up for drug intake. Chronotherapeutic drug release can be achieved by enteric film coating. Luo et al. [64] developed a fixed dose combination of telmisartan and pravastatin sodium in an enteric coated bilayer tablet that matched with circadian rhythmic variations of hypertension and cholesterol synthesis for once-daily bedtime dosing [64]. At first, bilayer tablets consisting of telmisartan and pravastatin sodium were seal-coated using Opadry^®^ to separate the core from the enteric coating polymers containing free carboxyl groups that might cause the degradation of pravastatin during coating or storage. Seal-coated bilayer tablets were finally enteric-coated using aqueous acrylic enteric polymer (Acryl-EZE^®^) [64]. These enteric-coated bilayer tablets prevented premature drug release in acidic gastric conditions and completely released the drug at pH 6.8. Such a delayed release drug delivery system with a bedtime dosage regimen is therapeutically recommended to match the circadian rhythmic variations of cholesterol synthesis and blood pressure. This system provides therapeutic benefits by maximizing therapeutic effects and patient compliance [64].

**Figure 3:**
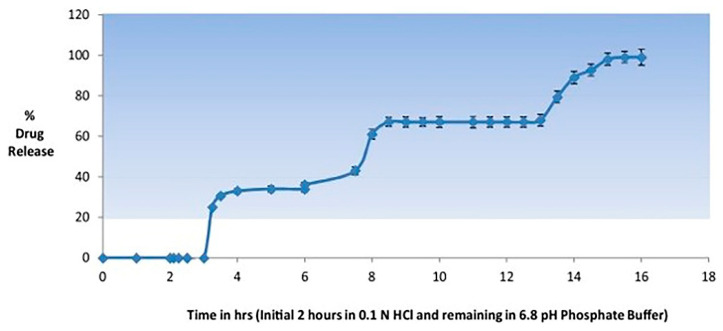
The in vitro drug release profile of enteric coated chrono-system with three pulsatile drug releases (adopted with permission from [65], Elsevier, 2016).

Jain and Devi [65] also developed a chronotherapeutic delivery system for *Gymnema sylvestre*, an antidiabetic herb. As diabetes requires a pulse of therapeutic concentration when blood glucose levels are high, they designed a chronotherapeutic drug delivery system to achieve three pulsatile drug releases from a single formulation within the circadian cycle. Three different formulations (immediate release tablets, delayed release coated tablets, and delayed release coated pellets) for time-controlled drug release were encapsulated into one capsule, which was finally enteric-coated with HPMC phthalate-50 to avoid the gastric mucosal membrane irritation and reflux [65]. An in vitro drug release study indicated three pulsatile drug releases from this system at fixed time intervals and gastric integrity for 2 h (Figure 3) [65]. Similarly, to counteract the morning symptoms of early morning pathologies, Cerciello et al. [66] developed a chronotherapeutic system for ketoprofen in which drug particles in pectin matrix were coated by Eudragit^®^ S100. This formulation achieved the delayed drug release via pH dependent solubility of the coating polymer, Eudragit S100^®^, and slow selective degradation of pectin matrix by intestinal microflora [66].

#### 4.1.2. Sustained Drug Release

Drug release rate can be controlled by physicochemical properties and the amount of polymers used for surface coating [67,68]. It is also controlled by altering the thickness, tortuosity, and permeability of the coating layer [69]. Coating materials for sustained drug release are usually water-insoluble and pH-independent, and are exemplified by ethyl cellulose, polyvinyl acetate, and polymethacrylate copolymers [68,70]. These polymers have good film forming properties and mechanical strength, making them suitable for sustained drug release coating [71]. The combined use of hydrophobic and hydrophilic polymers has also been attempted to optimize drug release. Venlafaxine HCl, an antidepressant, has a short half-life (about 5h) that requires a sustained-release formulation to reduce the dosing frequency. Jain et al. [72] developed a reservoir type aqueous and organic coated tablet of venlafaxine for sustained drug release using a typical ethylcellulose dispersion product (Aquacoat^®^ ECD 30) and polyacrylate-based coating agents (Eudragit^®^). Wan et al. [73] developed novel sustained release pellets of loxoprofen sodium via double-layered coating. These pellets consisted of a dissolution-rate controlling sublayer with hydroxypropyl methyl cellulose (HPMC) and citric acid (pH modifier) and an outer diffusion rate-controlling layer with aqueous dispersion of ethyl cellulose on the surface of a drug-loaded core pellet [73]. In vivo studies demonstrated that this double layered coating approach effectively achieved the prolonged systemic drug exposure [73].

**Figure 4:**
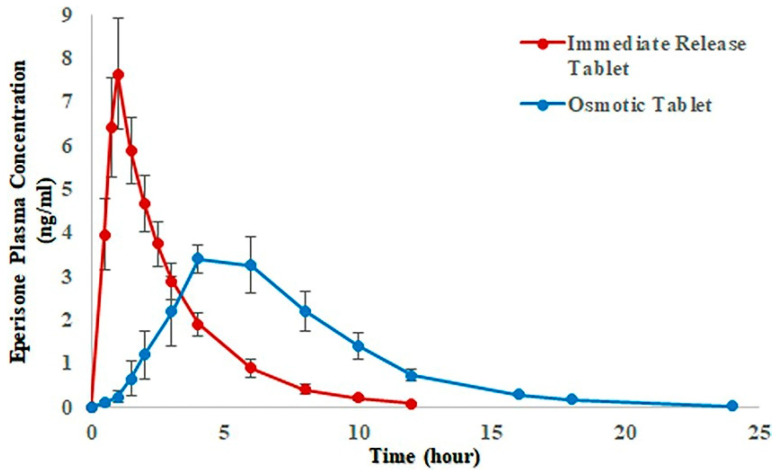
Mean plasma concentration versus time profiles obtained after administration of Eperisone 150 mg CR (controlled release) osmotic and immediate release tablets in 12 healthy subjects (Adapted from [77], Nature Research, 2020).

Surface coating approaches are also combined with different mechanism based-drug delivery systems to modify drug release rates. For example, osmotic pump delivery systems can be coated with water-insoluble polymers for sustained drug release. GI fluids diffuse through a polymer film and dissolve osmotic agents contained in the core, resulting in increased osmotic pressure and subsequent drug release through a laser-drilled orifice. Drug release can be controlled by orifice size and rate of fluid penetration, which depend on the thickness and permeability of the polymer film [70,71,74,75]. The polymer used for these systems must be sturdy enough to resist hydrostatic forces. Ethyl cellulose and cellulose acetate are the most commonly used polymers coating for osmotic pump systems [70,74]. Ahmed et al. [76] developed an osmotically controlled release (CR) formulation of eperisone hydrochloride in which core tablet was coated with Opadry^®^ CA (a cellulose acetate based-coating system) for a semipermeable thin film on the tablet. They conducted a comparative pharmacokinetic study of osmotically controlled release tablet and immediate release marketed tablet at a single dose of 150 mg eperisone in healthy human volunteers [77]. Results indicated the significant difference in Cmax and Tmax of two formulations (Figure 4). Compared to the immediate release tablet, the controlled release tablet extended drug release with a longer Tmax and a lower Cmax [77].

### 4.2. Improved Drug Stability

The stability of APIs or drug products can be altered by external environmental factors such as temperature, humidity, and light, as well as compatibility between excipients and APIs. Moisture can degrade drugs through hydrolysis and cause instability issues during storage [78]. Moisture-absorbed drug products can swell, crack, and dissolve inside the package, causing significant changes in product appearance and negatively affecting the shelf-life of the drug product [78]. Light also can stimulate oxidation and hydrolysis of APIs. To avoid these external factors causing instability of APIs or drug products, film coating can be applied to the surface of core tablets. Hydrophobic materials such as lipids, wax, and triglycerides are often used for sub-coating or outer-coating to protect the core tablet against external environmental factors [17]. Various water soluble and insoluble polymers, including polysaccharides, polypeptides, and vinyl polymers, are also widely used for film coating to improve drug stability [7].

The film coating approach used to improve the stability of drugs or drug products is exemplified as follows. Tablets containing light sensitive drugs (e.g., sorivudine, nifedipine, sulfisomidine, and molsidomine) undergo film coating for photostabilization [79]. Photostability mainly depends on the thickness of coating layers and can also be affected by concentration of the opacifier [80]. Film coating of core tablets has also been attempted to improve the product stability of moisture-sensitive drugs using various water-proof coating agents including polyvinyl alcohol (PVA), Eudragit^®^ EPO, hydroxypropyl methyl cellulose (HPMC), hydroxyethyl cellulose (HEC), and polyvinyl alcohol-polyethylene glycol (PVA-PEG copolymer). The effectiveness of moisture protective film coating depends on the type of polymers used and coating conditions. Combined use of different coating polymers at different proportions is also promising for improving the stability of moisture sensitive drugs. Heinamaki et al. [81] reported that inclusion of hydrophobic Suberin fatty acids (SFAs) isolated from outer birch bark in aqueous-based HPMC films significantly improved the water vapor barrier properties of the coating film. Similarly, ranitidine hydrochloride is hygroscopic in nature. Many efforts have been made to develop effective formulations and maintain product stability of ranitidine hydrochloride [82]. Patel et al. [83] reported that film coating using a combination of Eudragit^®^ EPO and Eudragit^®^ RLPO was effective in improving moisture stability and enhancing therapeutic efficacy of ranitidine hydrochloride [83].

### 4.3. Taste Masking

Unpleasant taste is a major hurdle to ensure patient compliance, particularly in pediatric and geriatric populations. Among various tastes, bitterness is the most repellent. Thus, masking bitter taste in oral dosage forms is a key parameter to improve patient compliance and therapeutic efficiency [7]. Optimized oral dosage forms should reliably hinder the release of bitter drug molecules in the mouth. However, taste masking should not negatively affect the bioavailability of drugs, or sensory awareness including mucosa irritation, roughness in the mouth, or hindered swallowing [84]. A variety of methods have been used for taste masking, such as chemical modification (prodrug approach), salt formation, interaction with ionogenic polymers (methacrylates), complexation, incorporation of flavor enhancers (e.g., sweeteners) in the formulation, and surface coating [7]. Among these methods, film coating is the most effective and commonly used approach for taste masking and is particularly suitable for microencapsulation of small particles to form taste masked multi-unit dosage forms [7,84]. Many different polymers are available for taste masking, including natural or synthetic polymers that hinder fast release of a bitter drug in the oral cavity and its contact with taste receptors in the tongue. In addition to water-soluble polymers, including starch derivatives, cellulose ethers, and hydrophilic block copolymers, water-insoluble polymers and gel-forming polymers can be also used to mask the taste [85]. These polymers are used alone or in combination with different polymers, preferably in combination with water-soluble and insoluble polymers at various ratios [7,85]. Nishiyama et al. [86] masked the bitter taste of lafutidine by film coating orally disintegrating tablets using a combination of water insoluble and soluble polymers (ethylcellulose and hypromellose). The polymer ratio in the taste-masking layer affected drug release lag time, drug release rate, tensile strength, and water permeability of the films [86].

### 4.4. Active Film Coating

Active film coating is a process to coat a solid dosage unit (tablet or pellet) using a solution or suspension containing APIs as a coating solution. This coating technology meets formulation needs such as rapid drug release or improved product stability and is particularly useful for developing fixed-dose combination (FDC) products to control the drug release rate or physically block interaction between APIs [87,88]. Water-soluble drugs can dissolve in aqueous coating solution or suspension that can then be sprayed on core tablets. Therefore, it is easier to develop an active coating process for water-soluble drugs than water-insoluble drugs [87]. With water-insoluble drugs, particle size must be fine enough to prevent clogging of spray guns. Moreover, the drug suspension should remain homogenous during the coating process to obtain satisfactory content uniformity [87].

Major challenges in active coating include (1) determining the coating end point to achieve target potency, (2) ensuring tablet-to-tablet API content uniformity, and (3) maximizing coating efficiency (ratio of amount of APIs deposited on core tablets to amount of APIs sprayed) [13,32,87]. During active film coating, tablets are periodically sampled and analyzed for weight gain as well as the amount of API deposited on core tablets using an in-process assay [88]. Based on this in-process assay, additional amounts of coating suspension are further sprayed until the coating end-point is reached to obtain the target potency. When coating conditions, especially spray rate, remain constant during the entire coating process, a linear relationship was observed between the actual API amount deposited on core tablets and coating time [13,32,87]. Control on the spraying operation is also important to ensure the content uniformity in active film coating. Content uniformity is affected by various process variables in the operation including spraying rate, inlet air temperature, residual moisture, pan speed, atomization pressure, and drug properties [13]. Therefore, it is important to understand the factors and coating mechanisms that impact content uniformity [87].

The API is directly mixed with the film-forming agent in active film coating, and there is no limitation in selection of the film-forming agent, including polyacrylates, polyvinyl alcohol, hypromellose, and hydroxypropyl cellulose [32]. Because APIs are present directly in the coating film, compatibility between film-forming agents and APIs should be confirmed. If necessary, a functional separation layer (e.g., enteric layer or hydrophobic layer) can be added between the core tablet and the active coating layer. Applications of active film coating are exemplified as follows.

**Figure 5:**
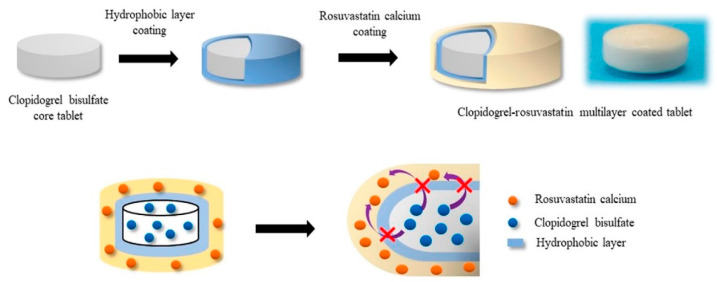
Structural illustration of the multilayer-coated tablet containing clopidogrel and rosuvastatin (Adapted from [91], MDPI, 2019).

Kim et al. [89] developed new fixed-dose combination tablets of metformin HCl and glimepiride using active film coating technology. As glimepiride is taken once daily and metformin HCl is taken twice daily, a new fixed-dose combination product for once daily dosing was developed by coating a glimepiride-immediate release (GLM-IR) layer on the extended-release core tablets of metformin HCl (MTF-ER). They also introduced an inert mid-layer to prevent contact between the MTF-ER core tablet and GLM-IR layer [89]. Common film-forming excipients, such as hypromellose, polyethylene glycol, and titanium dioxide, were used for the glimepiride coating layer, and sodium lauryl sulfate (SLS) was added to maintain a homogeneous glimepiride coating suspension [89]. Active film coating technology is also applied to enhance chemical stability of drugs. Desai et al. [90] explored an active film coating approach to stabilize peliglitazar, a PPAR α/γ agonist. As peliglitazar undergoes acid-/base-catalyzed degradation, the active film coating approach was applied by spraying drugs with coating materials onto a placebo core tablet to improve stability of the peliglitazar tablet formulation. This active film coating approach provided tablets with satisfactory chemical stability, which may be due to higher drug to excipient ratio in the film coat of non-reactive coating materials compared to traditional dry or wet granulated formulations [90]. Seo and Han [91] developed a stable and effective combination product of clopidogrel and rosuvastatin via active film coating. As illustrated in Figure 5 [91], the multilayer-coated tablet contained a hydrophobic separation layer between the clopidogrel core tablet and the rosuvastatin coating layer to avoid instability issues caused by direct mixing of rosuvastatin and clopidogrel acidic salt. The storage stability tests exhibited the good stability of both APIs in this combination product.

## 5. Recent Technology and Tools Used to Characterize Coated Tablets

As typical in vitro studies (e.g., dissolution studies) of optimization of coating conditions or process variables are often time-consuming, labor-intensive, and cost-ineffective, several technologies have been proposed as alternative methods. For example, multivariate image (MIA) and wavelet texture analysis (MWTA) can be used to characterize the coated tablets (e.g., color uniformity, surface roughness, and erosion). As they rely on simple color images from a digital camera, these techniques are easy, cost-effective, and feasible for industrial applications [92]. The electronic tongue is also a useful diagnostic technique to evaluate the taste-masking effect of film coated tablets, while the traditional human taste panel methods have issues associated with subjective assessment of tastes and there are difficulties interpreting results, particularly in pediatric formulations [93]. The electronic tongue is a device simulating human sense of taste that is composed of an array of chemical sensors and pattern recognition systems [93]. Advantages of this sensor array include relatively low cost of fabrication and analysis, possibility of analyzing small sample volumes, automation of analytical processes, and applications in on-line monitoring [93]. Similarly, SIGMI (SIMulator Gastro-Intestinal) and IntelliCap^®^ systems can be used to assess the effectiveness of film coating for modified drug release. While SIGMI is an automated in silico model, IntelliCap^®^ can be used for in vivo monitoring of the performance of coated formulations in the GI tract [2]. Previous studies have also demonstrated the gamma scintigraphy imaging technique as a helpful tool for visualizing the distribution of APIs in different segments of the GI tract [94,95].

Other non-destructive characterization techniques are also available, including nuclear magnetic resonance (NMR), attenuated total reflection Fourier-transform infrared spectroscopy (ATR-FTIR), confocal laser scanning microscopy, and terahertz pulsed imaging [96]. Ensslin et al. [97] used 1 H NMR to characterize the dissolution and release processes of drugs inside film-coated pellets. Feng et al. [98] used a time-dependent ATR-FTIR technique and two-dimensional correlation analysis to investigate the migration behavior of water and drugs across polymer films. Recently, Bernin et al. [99] used magnetic resonance imaging (MRI) to provide substantial and spatially resolved quantitative information about drug release through coating films. The underlying mechanisms, including differences in rate of water ingress, drug dissolution below the film, and hydrostatic pressure build-up triggering the onset of drug release, were clearly visualized by MRI [100].

Film coating of solid dosage forms is carried out using a range of equipment designs, process parameters, and coating formulations to manufacture the desired product. For pharmaceutical development and manufacturing of the desired coated product, it is important to choose an appropriate coating polymer formulation and to understand and optimize the complex coating process. Computational modeling of processes can aid in understanding, predicting, and troubleshooting film coating operations [100]. In addition, real-time assessment of coating quality is essential for automated production. The application of computational modeling and process analytical technologies for coated tablets is discussed below.

### 5.1. Computational and Mathematical Modeling of the Film Coating Process

Computational and mathematical models can be used to improve the mechanistic understanding of tablet coating processes and help to optimize the process [101]. Modeling approaches are useful to determine optimal process time, minimize the number of experiments, and reduce process failures, leading to lower development cost [102]. Various modeling systems have been proposed for film coating processes at different levels of complexity and predictive capability. Some models describe the coating phenomena at the surface of an individual tablet, while others predict mass coating uniformity for a batch of tablets being coated [100]. The latter include macro-level models such as phenomenological models and first-principle models. Phenomenological models (e.g., compartment models, Monte Carlo methods, and population balance models) describe the inherent variabilities in the coating process with probability distribution functions. They can be used to examine the coating processes with the variation of operating conditions. These models require one or more parameters to be measured experimentally [100,101,102,103]. On the other hand, first-principle models are exemplified by computational fluid dynamics (CFD) and discrete element method (DEM) [100,103]. These models describe the multiphase flow behavior in coating equipment. They may be used to predict the velocities of solid and gas, heat transfer within equipment, and temperatures of gas and solids phases [100,103]. In the case of DEM, every tablet in a coating pan is modeled using Newton’s laws of motion. The movement (position and velocity) of every tablet in the pan is calculated during the simulation. In addition, DEM can be used to investigate equipment design variables including number, size, and placement of baffles, nozzle geometry, and coating time [104]. Boehling et al. [102] set up a full-scale design of simulation experiment using DEM to examine the impact of various coating process parameters on the coefficient of inter-tablet coating variation (C_v,inter_), demonstrating that increasing the number of nozzles and decreasing the spray rate had the highest influence on C_v,inter_ [102]. Such models are extremely powerful due to their capabilities to predict a priori the impact of changes in operating variables on the quality of final product [28]. However, despite their predictive capability, these models are computationally intensive and require extremely long times of simulation for the coating process in industrial-sized equipment, limiting their real-time application. Nevertheless, these models are useful to understand the coating process and improve product quality. Development of more sophisticated and faster algorithms and increased computational power will facilitate their applications in pharmaceutical industry. On the other hand, micro-level models describe chemical and physical phenomena associated with film coating at the level of spray droplet-particle interaction, including atomization, droplet–particle collision, film formation, drying, and interparticle agglomeration [105]. These models are useful in determining the cause of morphological variations in coating properties.

Different modeling systems have different levels of complexity and predictive capability. Furthermore, the information obtained from different models is quite different. In certain cases, a combination of different models is needed for better understanding of coating process and troubleshooting. For example, Jiang et al. [106] developed a coupled CFD-DEM–Monte Carlo approach to predict intra- and interparticle film layer thickness and uniformity of porosity in a Wurster fluidized bed coater. The circulation motion of particles in the Wurster fluidized bed was simulated using CFD and DEM, while deposition, splashing, and drying droplets on the individual particle surface were modeled by the Monte Carlo method with spherical centroidal Voronoi tessellation [106]. The simulation results are compared with experimental data on residence time and intra-particle layer thickness distributions, indicating good agreement between the simulation and experimental measurements [106]. Several reviews of computational modeling of film-coating processes have been presented elsewhere [27,28,100].

### 5.2. Process Analytical Technology (PAT)

As the coating process is complex and influenced by many variables, real-time information on coating quality is essential for automated production. However, most industrial coating processes still depend on off-line measurements of critical quality attributes. In addition, the end-point of a coating process is usually defined by the amount of coating dispersion applied and the weight gain of coated dosage forms [107]. As these measurements provide little information on coating quality parameters (coating thickness, uniformity, density, etc.), they are unsuitable for predicting drug release behavior [108]. In recent years, the implementation of real-time process analysis technologies (PAT) has been actively pursued for more effective monitoring and controlling pharmaceutical unit operations. Particularly, non-destructive and noninvasive in-line measuring tools provide rapid feedback to allow the instantaneous process changes and control more effectively the critical quality attributes of final products [73].

Various PAT tools have been used to monitor the coating process of solid dosage forms and maintain the high quality of final products. These tools include spectroscopic techniques, imaging techniques, and microscopic techniques: (i) spectroscopic techniques include near-infrared spectrum (NIRS), Raman spectroscopy, and laser-induced breakdown spectroscopy (LIBS); (ii) imaging techniques include terahertz pulse imaging (TPI), near-infrared imaging, and magnetic resonance imaging (MRI); and (iii) microscopic techniques include confocal laser scanning microscope (CLSM), atomic force microscope (AFM), and scanning electron microscope (SEM) [109]. Among spectroscopic techniques, NIRS is one of the most widely used process analysis technologies; it is rapid, non-destructive, and cost-effective. NIRS can be used to analyze the coating thickness, coating end point, and coating uniformity. In addition, it can be used to predict drug release rate in combination with multivariate analysis [16]. NIRS has a disadvantage that the assignment of bands for more complex organic materials is complicated due to the absorption overlap of overtones and combination tones [110]. Raman spectroscopy examining the vibrational transitions in molecules is also applicable for quantitative analysis of the coating process. It can be used for real-time monitoring of drug polymorphic transformation and also determining drug contents, coating thickness, and coating uniformity [110]. Raman spectroscopy is a non-destructive method and requires little or no sample preparation. However, one major disadvantage of Raman spectroscopy is the inherently weak signal intensity, resulting in low sensitivity [110,111]. Furthermore, its application can be limited, particularly in the case of colored samples since interfering luminescence produced in many systems can mask the Raman spectrum [112]. Among imaging techniques, terahertz pulsed imaging (TPI) can be used to investigate the effect of coating equipment on the structure of applied film coatings and subsequent drug release performance [108]. This method allows for rapid image acquisition of samples of different shapes and sizes. It is a powerful tool for assessing pharmaceutical tablet coating quality and process control due to its high measurement precision [113,114]. In addition, it is a noninvasive analytical tool and does not cause thermal damage to the samples. However, it has disadvantages of high cost and low capacity [113,115]. Optical Coherence Tomography (OCT) is also increasingly applied to pharmaceutical film coatings, allowing fast and non-destructive analysis of coating thickness and quality via high-resolution cross-sectional images [116]. Sacher et al. [117] demonstrated the applicability of OCT in an industrial-scale pan coating process for real time monitoring of tablet coating quality (thickness, homogeneity, and roughness). OCT yields high-resolution images to assess both inter- and intra- tablet coating homogeneity and support active process control [117].

Collectively, PAT allows for real-time monitoring and data acquisition from a large amount of samples through the entire coating progress, providing more reliance on the quality of the final product [73].

## 6. Summary

Film coating is a common but critical process providing various functionalities to solid dosage forms, thereby meeting diverse therapeutic needs and increasing product values. Film coating is a technology-driven process, and the evolution of coated dosage forms relies on advancements in coating technology, equipment, analytical techniques, and coating materials. Many different coating technologies have been developed for solvent-based or solvent free coating processes. Each method has its own advantages and disadvantages and may require continuous technical refinement to ensure coating quality. In addition, advancements in coating equipment and materials are important to improve the film coating process and meet pharmaceutical needs. In the film coating process, intra- and inter-batch coating uniformity of tablets is critical to ensure the quality of the final product, especially for active film coating containing the active pharmaceutical ingredient in the coating layer. To reduce product failure and development cost, real-time assessment of coating quality is essential, particularly for automated production. Therefore, implementation of PAT is a recent trend for more effective monitoring and control of pharmaceutical unit operations. In addition to PAT, computational modeling is also actively pursued to aid understanding, predicting, and troubleshooting of film coating operations. Different modeling approaches have been proposed with different levels of complexity and predictive capability. Various process analytical techniques have been also developed for different levels of precision, capacity, and turnaround time. Each method has its own limitations and different predictive capability. The concerted efforts of conventional experiments, computer modeling, and real-time process analysis are necessary to save time and cost in developing the desired coated product. Given that digitalization is occurring across all manufacturing processes and is unavoidable in the coating sector, effective integration of big data generated from lab experiments, computational modeling, and in-line process analysis using technology such as artificial intelligence, machine learning, and natural language processing plays a critical role in moving forward to highly automated and digitalized coating processes in the near future.
